# Isolated high-altitude cerebral edema in the Himalayas: a case report

**DOI:** 10.1097/MS9.0000000000004199

**Published:** 2025-10-28

**Authors:** Rabin Paneru, Ranjeet Yadav, Sandeep Baishya, Suman Adhikari, Aashutosh Jha, Saurav Aryal, Subash Basnet, Mani Chandra Kandel

**Affiliations:** a Department of Radiology, Manipal College of Medical Sciences, Pokhara, Nepal; b Department of Internal Medicine, Gandaki Medical College, Pokhara, Nepal; c Department of Internal Medicine, Ascension Saint Agnes Hospital, Baltimore, USA; d Department of Dermatology, National Academy of Medical Sciences, Kathmandu, Nepal

**Keywords:** case report, cerebral edema, dexamethasone, high altitude, mountain sickness

## Abstract

**Introduction and importance:**

High-altitude cerebral edema (HACE) is the cerebral form of high-altitude sickness. It is a rare life-threatening condition that has been reported in 0.1–2% at altitudes above 3000 to 4000 m.

**Case presentation:**

In this case, the authors have presented a 47-year-old male who was rescued from the Everest Region of Nepal at an altitude of 4900 after complaining of a severe headache, followed by an episode of vomiting, along with confusion and inability to walk on his own. He was treated with dexamethasone and other supportive measures in the emergency department. Magnetic resonance imaging (MRI) findings were suggestive of HACE. The patient was subsequently admitted for observation and neurological consultation.

**Discussion:**

HACE can occur as a sequela of acute mountain sickness (AMS), concurrently with high-altitude pulmonary edema (HAPE), or in isolated form. The exact pathophysiology of HACE remains incompletely understood. Clinical features include severe headache, vomiting, dizziness, loss of consciousness, drowsiness, ataxia, and eventually lead to coma. MRI is the investigation of choice. Management includes immediate descent, supplemental oxygen, dexamethasone, and hyperbaric therapy.

**Conclusion:**

HACE has an unpredictable course, as it can progress rapidly. Proper acclimatization and taking appropriate precautions are essential when ascending to high altitudes.

## Introduction

High-altitude cerebral edema (HACE) is a rare, life-threatening condition occurring as the cerebral form of high-altitude illness. It can occur as the sequelae of acute mountain sickness (AMS), concurrently with high-altitude pulmonary edema (HAPE), or as isolated HACE^[1,2]^. High-altitude cerebral edema can occur in 0.1 to 2 percent at elevations above 3,000 to 4,000 meters whether mountaineers, sports personnel, hikers, or rescue workers if proper acclimatization is not taken while ascending^[1,3]^. Clinical features include nonspecific symptoms such as headache, dizziness, and lethargy, followed by a progressive decline in mental function and consciousness, along with cerebellar signs like ataxia, and eventually coma. However, HACE can occur acutely within hours without any symptoms of AMS^[2]^.

**HIGHLIGHTS**

- Isolated high-altitude cerebral edema (HACE) is rare, but can occur without preceding acute mountain sickness or pulmonary edema
- A 47-year-old male developed HACE, confirmed by MRI showing focal edema and microbleed.
- Prompt treatment with dexamethasone, supportive care led to full recovery.
- Early recognition, immediate descent, and prompt management are essential for favorable outcome.

Diagnosis of HACE is based on a clinical history, along with signs of encephalopathy, and is confirmed by typical findings in magnetic resonance imaging (MRI) of the head. Management includes immediate descent, oxygen therapy, isotonic crystalloids, acetazolamide, dexamethasone, and hyperbaric oxygen. Comatose patients require immediate airway protection along with neurological and neurosurgical interventions. Generally, pure cerebral edema is rare at altitudes below 5000 m, as it usually occurs in conjunction with pulmonary edema^[2,4]^. This case highlights a 47-year-old male from Singapore who was diagnosed with isolated HACE. He was rescued at an altitude of 4900 m above sea level from the Everest region and presented to our emergency department. This case has been reported in line with the Surgical CAse REport (SCARE) 2025 criteria^[5]^.

## Case presentation

A 47-year-old male from Singapore was brought to the Emergency Department (ED) of our hospital by helicopter rescue from Lobuche, which lies in the Everest region. He had a history of confusion, drowsiness, vomiting, severe headache, and inability to move without support. At Dingboche, which lies at an altitude of 4400 m, he had a cough and mild headache, which was not bothering him at all. Subsequently, he reached Lobuche at night, located at an altitude of 4900 m. Symptoms started to worsen overnight, and he started to experience a severe headache followed by an episode of vomiting, along with shortness of breath, confusion, and drowsiness. Additionally, he was unable to walk on his own. There was no history loss of consciousness, abnormal body movement, unrolling of eyes, frothing from mouth or bowel bladder incontinence. The minimum oxygen saturation measured was 71% in room air. He did not get oxygen support or any supportive medications as medical facility was not available at Lobuche. The next day, in morning, he was rescued by helicopter from Lobuche and brought to the ED of our center.

**Figure 1.**
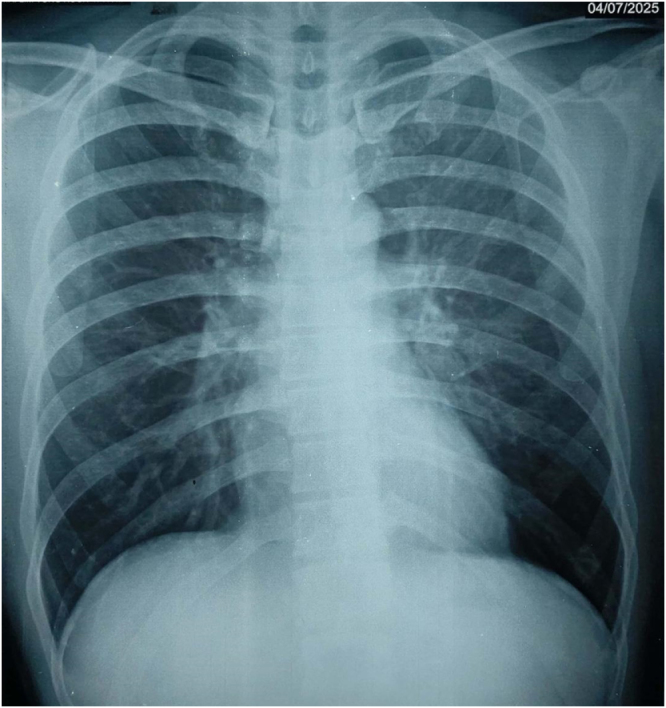
Normal chest radiograph of the patient performed at the time of presentation.

In the ED, he complained of a mild headache, nausea, and dizziness. He did not have any significant past medical, drug, or alcohol intake during the trek or trauma history. Vitals were stable: blood pressure 120/80 mmHg, heart rate 84 beats per minute (regular), respiratory rate 22 breaths per minute, and oxygen saturation 96% on RA. On examination, he was well-oriented to time, place, and person. GCS was 15/15. There was no ataxia or any other signs of focal neurological deficit. Respiratory examination did not reveal any signs of pulmonary edema. Other systemic examinations were normal. Workup in the ED included electrocardiogram, complete blood count, metabolic panel, coagulation studies, and chest radiograph. There were no lab abnormalities except for serum potassium, which was 3.4 milliequivalents/L. Chest X-ray showed clear lung fields which has been shown in Fig. 1.

**Figure 2.**
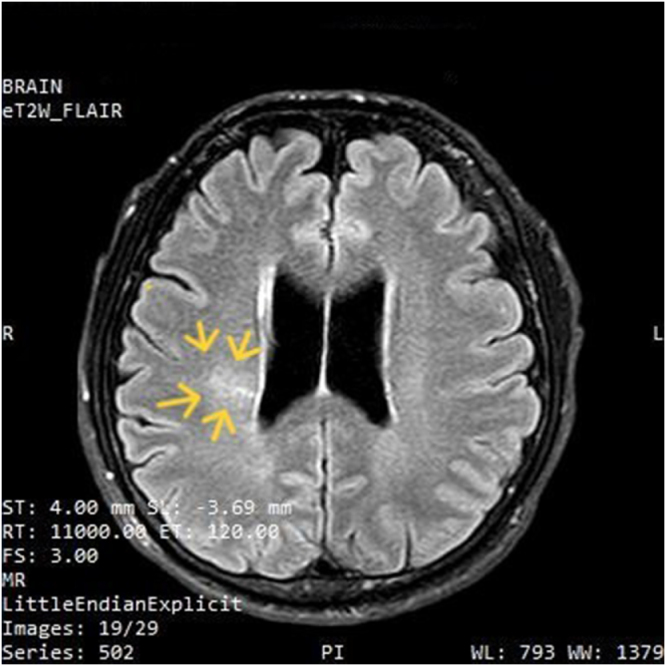
FLAIR image. Arrow heads showing focal area in the corona radiata of the right frontoparietal lobe that demonstrates band of high signal intensity suggestive of focal cerebral edema.

**Figure 3.**
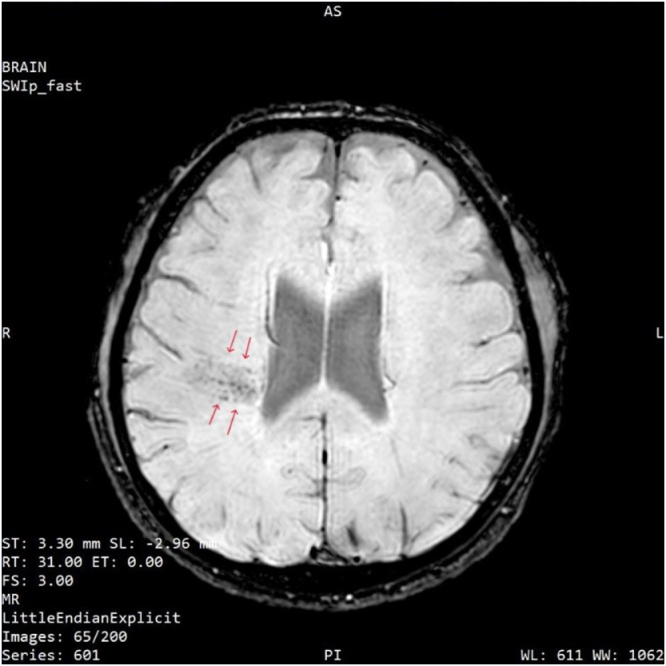
SWI sequence. Arrow heads showing foci of microbleeds.

At the ED, he was managed with intravenous (IV) dexamethasone 8 mg initial dose followed by 4 mg every 6 hourly^[6]^, IV fluid normal saline, IV ondansetron 4 mg, and tablet paracetamol. Subsequently, he was planned for MRI of the head. MRI revealed a focal area in the corona radiata of the right frontoparietal lobe that demonstrates a band of high signal on fluid-attenuated inversion recovery (FLAIR) image (shown in Fig. 2) and foci of microbleeds on susceptibility-weighted imaging (SWI) sequence (shown in Fig. 3).

Subsequently, the diagnosis of high-altitude cerebral edema (HACE) was confirmed. The patient was admitted to the ward for observation and neurology consultation. His serum electrolyte was repeated on the next day, and all the parameters were within normal limits. There were no features suggestive of raised intracranial pressure (ICP). On the fourth day of admission, his symptoms completely resolved except for a mild headache. Intravenous fluids, dexamethasone, and ondansetron were stopped except for oral paracetamol tablets. He was discharged on the fifth day after being properly counseled regarding the future high-altitude expeditions and danger signs and symptoms of HACE.

## Discussion

HACE can occur in individuals of any age, whether male or female. Young population, especially males, may be at high risk because they tend to ascend despite experiencing symptoms of AMS^[7]^. There are no physiological, anatomical, or genetic markers indicating susceptibility to HACE. However, it has been found that persons with pre-existing elevated intracranial pressure, vascular malformation, multiple sclerosis, cerebral venous thrombosis, intracranial hemorrhage, hydrocephalus, intracranial space-occupying lesions, and other neurologic conditions are at increased risk for HACE^[8]^. Our patient did not have any predisposing factors for developing HACE.

HACE has been reported at altitudes as low as 2100 m, although it is more commonly found at elevations above 3000 to 4000 m^[2,4]^. It has been found that among patients diagnosed with HACE, HAPE has been commonly reported. Also, it has been reported that 50% of patients who die from HAPE also suffer from HACE^[9]^. In a similar case published by Chen *et al*, they present a 23-year-old male who developed HACE and HAPE in Himalayan region at an altitude of 3656 m^[10]^. In another case published by Bolotin *et al*, they report a 27-year-old male with isolated HACE at mere height of less than 3000 m^[11]^. In our case, the patient was a young male, had no previous known risk factors, and had starting exhibiting some symptoms at 4400 m, and developed isolated HACE at 4900 m.

Hypoxemia leading to cerebral vasodilation is the initial event in the development of AMS^[12,13]^. Vasodilation further leads to an increase in brain volume, decreased compliance, followed by raised ICP. Although AMS typically resolves within 2–3 days of acclimatization, those who develop HACE follow a cascade of edema^[8]^. Though AMS represents a sequela of HACE, their tendency to share a similar underlying pathophysiology is not clearly understood. Cerebral edema due to the accumulation of water in the brain parenchyma, including the interstitial and intracellular compartment, is consistently found in the MRI of patients with clinical HACE and severe AMS^[14–16]^. In our case, MRI showed a focal lesion in the right frontoparietal corona radiata with a high signal band on FLAIR and microbleeds on SWI.

There are three types of brain edema: intracellular (or cytotoxic), ionic, and vasogenic^[14].^ Vasogenic edema may lead to worsening of symptoms, loss of white matter vasculature, microbleeds, and hemosiderin deposition^[4,17].^ Generally, computerized tomography (CT) is normal at an initial phase, therefore, we directly performed an MRI. After progression into ionic edema, loss of gray–white matter differentiation is detectable in the CT scan^[18]^. In MRI, ionic edema can be detected as hyperintensity on T2-WI or FLAIR images. Both white and gray matter are usually affected with restricted diffusion on diffusion-weighted image (DWI) and reduced signal on apparent diffusion coefficient (ADC)^[19]^. Similarly, in vasogenic edema, there is hyperintensity on T2-WI and FLAIR images but without restriction on DWI-WI^[14]^. In susceptibility-weighted neuroimaging (SWI), microbleeds are found throughout the cerebral white matter tracts in HACE survivors, whereas they are absent in AMS and isolated HAPE^[4,20]^.

Initially, HACE may be mistaken for exhaustion, as it can present with irritability, lethargy, and apathy for activities. Other features may include confusion, ataxia, drowsiness, and finally, coma. It can progress unpredictably above 3000 m, and may occur in as little as 12 h. Signs of abnormal coordination, such as impaired performance of finger-to-nose and heel-to-toe walking tests, may be present. Focal neurological deficits, such as hemiparesis or distinct visual disturbances, should raise concern for alternative diagnoses, as they are uncommon^[21,22]^. HACE can present concurrently with HAPE; as a result, pulse oximetry shows hypoxemia^[23,24]^. Crackles may present with pulmonary edema on chest X-ray. CSF shows normal count with an increase in opening pressure^[17]^. For instance, in our case, the patient’s mild symptoms at 4400 m worsened after ascending to 4900 m, progressing rapidly to severe headache, vomiting, confusion, and loss of mobility, and on respiratory examinations, lung fields were clear to auscultation and chest radiography.

Diagnosis of HACE is made based on the history of ascent above an altitude of 3000 m with concurrent signs of encephalopathy. Definitive diagnosis is made based on MRI findings, but treatment should be started promptly. MRI studies reveal characteristic increases in T2 and FLAIR signal in the splenium of the corpus callosum and subcortical white matter with no gray matter lesion^[3]^. In fatal cases, petechial hemorrhages and thromboses, including venous sinuses, are demonstrated in MRI^[25,26]^. The severity of edema cannot be correlated with clinical outcome^[9]^. MRI findings may persist for days or weeks following recovery, whereas hemosiderin deposition can last for years^[4]^. In our case, MRI suggested focal cerebral edema and microvascular hemorrhage. A follow-up MRI was not deemed necessary due to the patient’s clinical improvement.

Early recognition of the signs and symptoms of HACE is critical, as immediate intervention is required. Immediate descent is crucial when HACE is suspected, especially in the presence of early features like ataxia and deterioration in mental function. A descent of 1000 m has been reported to be typically lifesaving. If immediate descent is not possible, dexamethasone, supplemental oxygen, and hyperbaric therapy should be considered until evacuation, as these measures help to temporize the illness^[2,27]^. Dexamethasone, at an initial dose of 8 mg, is administered orally, intramuscularly (IM), or intravenously (IV), until descent is achieved, and is effective^[6]^. However, symptoms may recur if the medication is discontinued or if acclimatization has not been achieved^[28]^. Hyperbaric therapy is also effective if immediate descent cannot be achieved, but it should be combined with dexamethasone and supplemental oxygen^[29,30]^. A patient in a coma despite the appropriate measures mentioned above requires immediate admission to the intensive care unit (ICU) for ICP monitoring^[27]^. In addition, other causes should also be thoroughly assessed. The patient may require weeks to recover, and severe cases result in death despite interventions^[16].^ Permanent neurological impairment has also been reported in survivors^[16,31]^. Rapid descent probably halted the progression of cerebral edema in our patient, and symptoms did not recur after admission, and further investigations were not done based on clinical judgement. Furthermore, we could not assess for the long-term sequalae of our patient.

Acetazolamide is used as a prophylactic agent for the prevention of AMS but is not effective against HACE. Ascent with proper acclimatization and immediate descent following suspicion is essential to prevent HACE. Our case is a classic yet underreported clinical progression of isolated HACE, who was managed with dexamethasone, and supportive measures, without any residual complications. This case demonstrates the potential of full neurological recovery if the patient can be brought promptly in an appropriate treatment center. This case also highlights the unpredictability of altitude sickness, where mild, seemingly benign symptoms can progress into a life-threatening cerebral edema during ascent.

## Conclusion

HACE is a life-threatening condition that can occur without any symptoms of AMS or HAPE at high altitude. It is unpredictable in its progress. Even mild symptoms of AMS at altitudes above 3000 to 4000 m should be anticipated as a progression to HACE. Prompt descent upon recognizing the signs and symptoms of HACE is critical and can be life-saving. Furthermore, for individuals at moderate to high risk for developing acute mountain sickness, a prophylactic dose of acetazolamide 125 mg every 12 h should be started the day before ascent and continued 2–3 days at maximum altitude^[6]^.

### Limitations

Subsequent follow up after discharge was not possible, as the patient returned to his home country and could not be contacted, which limited our understanding of postdischarge recovery.

## Data Availability

Available upon reasonable request.
